# When arousal meets cognitive load: affective arousal and germane processing in a Stroop-like task

**DOI:** 10.3389/fpsyg.2026.1804728

**Published:** 2026-05-13

**Authors:** Susanne Koerber, Babette Park

**Affiliations:** Department of Psychology, University of Education Freiburg, Freiburg, Germany

**Keywords:** affective arousal, attentional control, cognitive load theory, cognitive resource allocation, germane processing, Stroop-loke task

## Abstract

This study investigates how affective arousal relates to distinct components of cognitive load during a cognitively demanding computer-based task. Affective arousal was measured pre- and post-performance using a brief pictorial instrument (POET-A), capturing momentary activation states in a language-independent format. Intrinsic, extraneous, and germane cognitive load were assessed following performance in a Stroop-like task in 120 participants (*M* = 23.72 years, SD = 5.72; 103 women, 15 men, 2 diverse), with germane cognitive load used as an indicator of germane processing. Results showed a consistent positive relation between affective arousal (pre- and post-task) and germane cognitive load, indicating an association with strategic, task-related cognitive processing. In contrast, affective arousal showed no (pre-performance) and only weak negative (post-performance) relations with intrinsic and extraneous cognitive load, underscoring the functional specificity of these associations. Germane cognitive load was additionally related to Stroop-like task performance, particularly to process-sensitive performance indices (reaction time), whereas affective arousal itself was not directly associated with task performance. These findings show a systematic and selective association between affective arousal and germane processing, highlighting its potential role in cognitive resource allocation during task engagement within a Cognitive Load Theory framework.

## 1 Introduction

Cognitive Load Theory (CLT) provides a well-established framework for understanding how limitations of working memory constrain learning and problem solving (Sweller, 1988). A central assumption of CLT is that learning outcomes depend not only on the amount of information presented but critically on how limited cognitive resources are allocated during task processing (Paas et al., 2003a). When cognitive demands exceed available resources, learning is impaired; when resources are efficiently allocated, deeper and more durable learning becomes possible (van Gog and Paas, 2008).

To capture these dynamics, CLT distinguishes between three types of cognitive load. Intrinsic cognitive load reflects the inherent complexity of the task material and is primarily determined by element interactivity and prior knowledge (Sweller et al., 2011). This theoretical characterization is mirrored in experimental work showing systematic relations between task complexity, intrinsic-load ratings, and perceived task difficulty

(Leppink et al., 2014). Extraneous cognitive load arises from suboptimal instructional design and unnecessary processing demands, such as split-attention or redundancy effects, which have been shown to induce processing inefficiencies (Chandler and Sweller, 1991; Kalyuga, 2011). In contrast, germane cognitive load refers to the allocation of cognitive resources to learning-relevant processing and schema construction and is only weakly related to task difficulty but systematically associated with strategic engagement and learning outcomes beyond difficulty alone (Eysink et al., 2009; van Gog and Paas, 2008). This differentiation is also reflected in factor-analytic studies of subjective load measures, which distinguish between intrinsic, extraneous, and germane components (Klepsch et al., 2017; Leppink et al., 2013). While CLT provides a well-established framework for understanding how cognitive resources are structured and constrained, it remains largely silent on how momentary learner states shape the allocation of cognitive resources during task engagement. The present study addresses this gap by examining affective arousal as a momentary activation state that may systematically influence how cognitive resources are allocated during task engagement.

### 1.1 From germane load to germane processing

Within CLT, the germane component of cognitive load has traditionally been described as the cognitive resources devoted to schema construction and learning-relevant processing (Sweller, 1988; Sweller et al., 2011). Early formulations treated germane load as an additive component, conceptually distinct from intrinsic and extraneous load representing the portion of cognitive effort that directly benefits learning (Paas et al., 2003b).

More recent theoretical work, however, has refined this interpretation by emphasizing that the germane component of cognitive load does not constitute an additional burden on working memory, but rather reflects how learners actively invest available cognitive resources during task engagement (Sweller, 2010; Sweller et al., 2019). Accordingly, germane load has been reframed as germane processing, focusing on the quality of learners' cognitive engagement, including sustained attention, strategic regulation, and effortful integration, rather than on load quantity. Conceptually, this process-oriented view aligns with critiques of additive load models that emphasize qualitative differences in cognitive processing (Schnotz and Kürschner, 2007). Empirically, this interpretation of germane processing is supported by evidence showing that germane related measures are only weakly related to perceived task difficulty, but strongly linked to strategic engagement, self-regulated learning, and deeper learning outcomes (de Koning et al., 2011; van Gog and Paas, 2008).

This reconceptualization has implications for how learning-related cognitive processes are studied. Whereas intrinsic and extraneous load are largely determined by task characteristics and instructional design, germane processing is inherently dynamic and context-sensitive, varying as a function of learners' momentary engagement, attentional control, and strategic investment. In the present study, the germane component of cognitive load is therefore conceptualized in process-oriented terms, capturing learners' active cognitive engagement during task execution rather than retrospective evaluations of learning outcomes.

### 1.2 Affective arousal as a modulator of cognitive processing

Arousal is a central construct in accounts of cognition and affect, yet its meaning varies across theoretical traditions. While classical approaches have emphasized physiological activation and neurocognitive models have highlighted the role of neuromodulatory systems in regulating attention (Aston-Jones and Cohen, 2005), more recent perspectives converge on the idea that what matters for cognitive performance is not activation *per se*, but how it is experienced and functionally deployed during task engagement. Rather than adopting any single tradition, the present approach integrates these perspectives by linking neurocognitive accounts of gain modulation with dimensional models of affect, thereby conceptualizing arousal as an experiential activation state that is functionally relevant for cognitive control.

In line with dimensional models of affect (Russell, 1980), arousal is understood here as the activation component of core affect. The term *affective arousal* is used to emphasize that this activation is embedded in affective experience, while remaining distinct from both valence and discrete emotional states. Within this framework, arousal reflects a subjectively experienced continuum ranging from low activation (e.g., fatigue, low alertness) to heightened activation (e.g., alertness, tension, or even overstimulation).

Building on this view, we conceptualize arousal as a dynamic activation state that reflects momentary task readiness and is assumed to shape how cognitive resources are allocated during task engagement. From this perspective, arousal is not assumed to directly enhance or impair performance, but rather to influence how efficiently attention is stabilized and directed toward task-relevant information.

Empirical and theoretical work suggests that this modulation operates in a non-linear manner, consistent with classic inverted-U accounts of arousal and performance (Yerkes and Dodson, 1908). Relatively low levels of activation are associated with reduced engagement, whereas excessively high levels can destabilize cognitive control, particularly under conditions of interference (Arnsten, 2009; Eysenck, 2012). Neurocognitive accounts provide a mechanistic interpretation of this pattern, proposing that arousal-related neuromodulatory activity regulates gain in cortical processing and thereby biases the competition between task-relevant and task-irrelevant information (Aston-Jones and Cohen, 2005; Mather and Sutherland, 2011). This attentional modulation can be understood as a mechanism through which arousal shapes the conditions under which cognitive control operates.

This perspective is particularly relevant for tasks requiring selective attentional control under interference, such as Stroop-like paradigms (MacLeod, 1991). Successful performance depends on maintaining task-relevant processing in the presence of competing stimulus dimensions. Variations in arousal are therefore expected to influence not only overall performance, but specifically the efficiency of attentional selection and interference resolution.

Thus, affective arousal is treated as an activation state rather than as cognitive processing itself and is therefore theoretically distinct from attentional control and germane processing, which reflect the regulation and allocation of cognitive resources during task engagement. Accordingly, the present study focuses on subjectively experienced arousal as a dynamic state variable capturing fluctuations in alertness, tension, and task-related activation, rather than physiological arousal indices or discrete emotional states, without reducing it to undifferentiated general arousal. This operationalization is intended to capture the experiential aspect of activation that is most directly linked to cognitive engagement.

### 1.3 Why affective arousal should selectively relate to germane processing

Against this background, given that affective arousal is assumed to influence attentional control during task engagement, its effects are expected to be specific to germane processing rather than general across load components. Intrinsic cognitive load is primarily determined by task-immanent structural characteristics, whereas extraneous cognitive load arises from design-induced, unnecessary processing demands. Germane processing, in contrast, is learner-driven and reflects the extent to which available cognitive resources are invested in sustained attention, strategic regulation, and effortful task engagement (Sweller, 2010), and is therefore closely linked to the stability and regulation of attentional control. As such, it directly captures how cognitive resources are deployed during task execution. From this perspective, any influence of affective arousal on task processing should be most evident in germane processing, as this component most directly reflects the investment of cognitive resources.

### 1.4 Affective arousal in cognitive load research: empirical gaps

While prior research within CLT has increasingly considered emotional and motivational factors (e.g., Kalyuga and Plass, 2025; Mayer, 2021; Park et al., 2015; Plass and Kalyuga, 2019; Um et al., 2012), these influences are typically examined as broader affective constructs rather than as specific activation states. More specifically, emotion-related research within CLT has primarily linked emotional influences to extraneous cognitive load, focusing on emotionally charged or aesthetically enriched materials that increase processing demands unrelated to learning (Plass and Kalyuga, 2019). This line of research therefore predominantly frames affective influences as additional sources of cognitive demand rather than as factors that may shape cognitive engagement.

Given evidence from related domains linking arousal to attentional control (e.g., Aston-Jones and Cohen, 2005; Mather and Sutherland, 2011), incorporating a focus on affective arousal as a specific activation dimension into CLT research may provide a more fine-grained account of how cognitive resources are deployed during task processing. Taken together, this literature underscores the need to move beyond treating emotional and broader affective influences as general sources of disturbance or additional cognitive load and instead to consider how they may shape cognitive engagement within CLT.

To examine these mechanisms in a controlled setting, tasks requiring selective attentional control under interference are particularly informative. Stroop-like paradigms provide a well-established means of assessing attentional selection in the presence of competing information (MacLeod, 1991). Although such tasks do not directly assess learning outcomes, they are sensitive to variations in attentional control and thus provide an appropriate context for examining how differences in cognitive resource investment are reflected in task performance.

### 1.5 The present study

The present study examines how affective arousal relates to the allocation of cognitive resources during engagement with a computer-based task, drawing on CLT and a process-oriented conceptualization of germane processing. From an affective resource allocation perspective, arousal is conceptualized as an activation state that is assumed to shape how available cognitive resources are invested during task execution. Accordingly, affective arousal is assumed to influence the efficiency and direction of cognitive engagement, particularly with respect to germane processing.

Affective arousal was assessed using POET-A, a newly developed brief pictorial measure capturing engagement-related arousal in a language-independent format. It was subsequently recoded along a graded dimension of functional optimality, reflecting the extent to which arousal approximated levels conducive to effective cognitive engagement (Aston-Jones and Cohen, 2005). Cognitive load was measured using subjective ratings differentiating intrinsic, extraneous, and germane components, with germane items capturing cognitive engagement during task execution. Task performance was measured with a computer-based Stroop-like task. Although affective arousal and cognitive load are conceptually distinct, both constructs are typically assessed using subjective self-report measures. It is therefore important to examine whether arousal represents a construct that is empirically distinguishable from established cognitive load components within this measurement context.

The study addresses two research questions: (1) whether affective arousal constitutes a construct distinct from established components of cognitive load, and (2) whether affective arousal is selectively associated with germane processing rather than with intrinsic or extraneous cognitive load, including its predictive relation to germane processing.

## 2 Methods

### 2.1 Participants

Participants were 120 university students (*M* = 23.72 years; SD = 5.72 years; 103 women, 15 men, 2 non-binary), recruited from a midsized city in southern Germany. All participants provided informed consent by completing the online questionnaire, following the Declaration of Helsinki.

### 2.2 Measures

#### 2.2.1 Assessment of affective arousal

**Figure 1:**
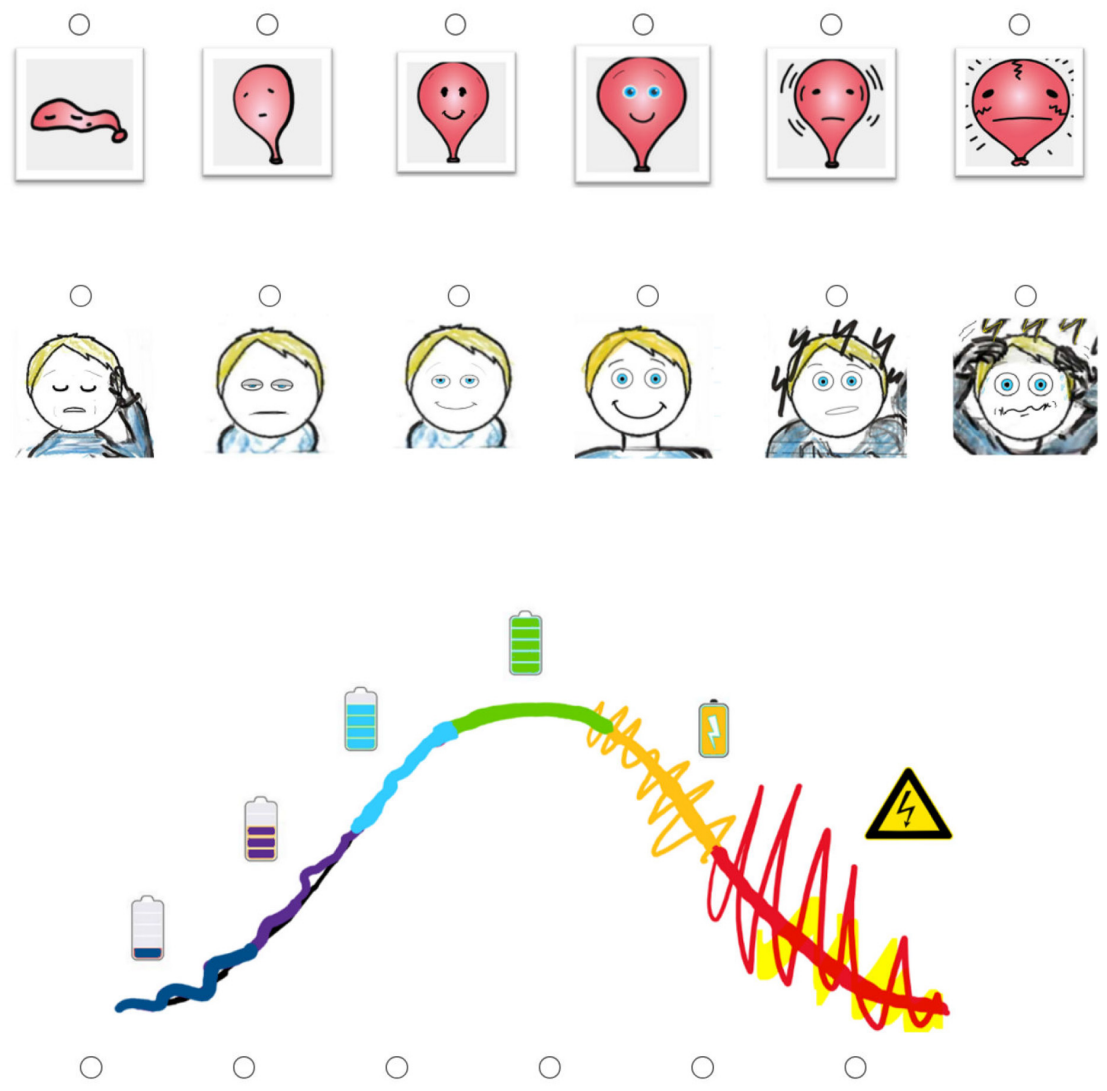
Three items of the POET-A scale illustrating increasing levels of affective arousal from extremely low to extremely high activation.

*Pictorial affective arousal assessment (POET-A)*. Affective arousal was assessed using a brief three-item pictorial measure (POET-A) each consisting of six-level Likert-type scales depicting a balloon, a face, and a curve (Figure 1). The pictorial format was chosen to provide a brief and intuitive measure of subjectively experienced activation that can be administered repeatedly during task performance without imposing additional verbal or cognitive demands. To support the construct validity of the measure, established verbal arousal ratings were included and used as convergent indicators of subjective activation. Each image represented increasing levels of affective arousal, ranging from minimal (extremely low) activation to maximal overactivation. For example, the balloon illustration ranged from deflated to overinflated, with two intermediate images representing functionally optimal arousal states, depicting a relaxed but engaged state and a fully awake, attentive state.

Participants rated their current affective arousal at pre- and post-task time points surrounding a Stroop-like task. Responses were analyzed using two complementary scoring approaches. First, ratings were treated as continuous scores (1–6) reflecting the perceived intensity of arousal from minimal to maximal activation. Second, responses were recoded into a three-level functional arousal index, reflecting proximity to an optimal arousal state in line with inverted-U-shaped accounts of arousal–performance relations (e.g., Yerkes and Dodson, 1908) and grounded in contemporary models of arousal-related performance regulation (e.g., Aston-Jones and Cohen, 2005). In this coding scheme, which was used as the primary arousal index for all analyses, 0 indicated clearly suboptimal arousal (marked under- or over-arousal), 1 indicated moderately suboptimal arousal (near-optimal but still under- or over-activated), and 2 indicated optimal arousal (relaxed or wide awake).

*Verbal affective arousal assessment (verbal arousal rating):* In addition to the pictorial scales, affective arousal was assessed post-task using a verbal six-point Likert scale. Participants rated their current state ranging from 1 (“completely drained”/“total schlapp”), 2 (“a bit tired”/“etwas müde”), 3 (“relaxed”/“entspannt”), 4 (“wide awake”/“hellwach”), 5 (“tense”/“angespannt”), to 6 (“overstimulated”/“total überspannt”). Verbal ratings were scored both as continuous values (1–6) and recoded into the same three-level functional arousal index (0–2) used for the pictorial measures.

*PANAVA_Arousal*. Participants completed a shortened version of the PANAVA-KS scale (Schallberger, 2005; see also Knörzer et al., 2016), a seven-point bipolar Likert measure of momentary emotional state. For this study, three items primarily reflecting arousal with minimal valence content were selected based on consensus of three experts: (1) energy level (“without energy – full of energy”), (2) tiredness (“tired – wide awake”), and (3) stress (“stressed – relaxed”). These items were coded so that higher scores indicated movement from under- or over-arousal toward an optimal, energized state, and then combined into an arousal subscale to establish the external validity of the pictorial affective arousal measures (POET-A).

Together, these measures are intended to capture subjectively experienced activation states (e.g., alertness, tension, and fatigue) as conceptualized within core affect, rather than physiological arousal or discrete emotional states.

#### 2.2.2 Cognitive load measures

Cognitive load was assessed with a 9-item, 9-level Likert scale. Each subdimension comprised three items. Intrinsic and extraneous cognitive load were measured with items closely aligned with the questionnaire proposed by Leppink et al. (2013), with wording adjustments to reflect the task-based and non-instructional nature of the Stroop paradigm, while preserving the original conceptual meaning of the items.

Intrinsic load items captured perceived task complexity and the cognitive demands arising from this complexity, including effort invested due to intrinsic task characteristics. Extraneous load items addressed clarity and effectiveness of task instructions and presentation, capturing unnecessary cognitive effort resulting from suboptimal instructional or representational features. In contrast, the germane component was operationalized using a process-oriented approach focusing on participants' subjective concentration, sustained attentional engagement, and strategy-related cognitive engagement during task execution. This operationalization builds on Salomon's (1984) conceptualization of invested mental effort and aligns with contemporary process-oriented perspectives on germane processing (e.g., Sweller et al., 2011; see also Klepsch et al., 2017). One strategy-related item was included to capture a regulatory facet of cognitive engagement that may support germane processing in complex, interference-rich tasks, complementing attentional engagement assessed by the other germane items. A full list of items and their adaptations is provided in Table 1.

**Table 1:** Adapted cognitive load items for the Stroop-like task.

No.	Dimension	Adapted item (English/German)	Reference item (Leppink et al.)	Comment on adaptation
1	Intrinsic	These tasks were very complex. Diese Aufgaben waren sehr komplex.	The topic/topics covered in the activity was/were very complex. (App. 1, il1)	Wording adapted to task context; conceptual equivalence preserved.
2	Intrinsic	The complexity of these tasks required a lot of mental effort. Ich habe sehr viel mentale Anstrengung in die Komplexität dieser Aufgaben investiert.	The activity covered formulas that I perceived as very complex. (App. 1, il2).	Emphasizes experienced mental effort resulting from task complexity.
3	Intrinsic	Tasks that required me to hold two things in mind at the same time were difficult. Aufgaben, bei denen ich zwei Sachen auf einmal im Kopf behalten muss, fallen mir eher schwer.	—	New item capturing element interactivity and simultaneous processing demands (cf. Klepsch et al.)
4	Extraneous	The explanations for these tasks were very unclear. Die Erklärungen zu diesen Aufgaben waren sehr unklar.	The instructions and/or explanations during the activity were very unclear. (App. 1, el1)	Minor wording change; conceptual equivalence preserved.
5	Extraneous	Unclear explanations required a lot of mental effort. Mich haben unklare Erklärungen zu dieser Aufgabe viel mentale Anstrengung gekostet.	The instructions and/or explanations were, in terms of learning, very ineffective. (App. 1, el2)	Emphasizes mental effort due to ineffective explanations.
6	Extraneous	The task itself was clearly presented. Die Aufgabe selbst war übersichtlich präsentiert.	The instructions and/or explanations were full of unclear language. (App. 1, el3).	Assesses clarity of task presentation; reverse-coded.
7	Germane	I was able to maintain focus on the task well. Ich konnte den Fokus auf die Aufgabe gut halten.		New item capturing sustained attentional engagement during task execution.
8	Germane	I was good at concentrating on the task. Es gelang mir gut, mich auf die Aufgabe zu konzentrieren.	How much did you concentrate during the lecture? (App. 2, Item 4)	Adapted process-oriented concen-tration item originally proposed by Salomon (1984) and used by Leppink et al.
9	Germane	I have good strategies to work through such tasks. Ich habe gute Strategien, mit denen ich solche Aufgaben bearbeite.	-	New item capturing strategy-related cognitive engagement (cf. Klepsch et al.)

Intrinsic and extraneous load items were adapted from Leppink et al. (2013). Germane processing items reflect attentional and strategy-related engagement. All items were rated on a 1–9 scale.

Throughout the manuscript, “germane cognitive load” refers to the measurement scale, whereas the underlying construct is conceptualized as germane processing, reflecting the allocation of cognitive resources during task engagement. Engagement-related constructs are acknowledged as conceptually related, reflecting observable aspects of how cognitive resources are allocated during task engagement.

#### 2.2.3 Performance test

Cognitive performance was assessed using a 14-item Stroop-like task designed to measure selective attentional control under interference, a core executive control process relevant for managing competing stimulus representations. In line with the classic Stroop paradigm, color words (black, blue, red, green, orange, and pink) were presented in incongruent ink colors. Participants were required to identify the single target word whose meaning matched its ink color (e.g., “blue” printed in blue ink) while ignoring distractor words. Responses were made via mouse click. The first two items displayed five words in a single row; the remaining 12 items presented ten words arranged across two rows. The words were presented simultaneously on screen, with one target item and multiple distractors differing in color–word congruency. Each trial advanced immediately after a response was made, resulting in a self-paced procedure. Participants were instructed to respond as quickly and accurately as possible. In each trial, the target word was randomly positioned, color–word combinations were varied across trials, and stimuli were presented in randomized order across participants. Prior to the test, participants received standardized instructions and completed a practice item to ensure task comprehension. The test commenced only after successful completion of the practice trial.

As indicators of performance, (1) the mean reaction time (Stroop mean RT, in ms, recorded automatically by the computer system) and (2) a performance efficiency score, calculated by dividing the number of correct responses by the participant's mean RT (Stroop efficiency), were computed. Reaction time served as a process-sensitive index of selective attentional control under interference, whereas the efficiency score provided an integrated indicator of overall task performance combining speed and accuracy.

### 2.3 Procedure

Participants completed an online questionnaire lasting approximately 10–15 min. Pre-task affective arousal was assessed using the pictorial affective arousal assessment (POET-A), followed by the PANAVA arousal scale. Participants then completed the Stroop-like performance task. Immediately after task completion, cognitive load was assessed using the adapted nine-item cognitive load scale measuring intrinsic, extraneous, and germane load. Finally, post-task affective arousal was reassessed using both the POET-A and a verbal arousal rating. The study employed a pre–post design focusing on inter-individual differences within a single, standardized task. Task characteristics were not systematically varied, as the focus of the analyses was on variability in learner states rather than on within-task variation in cognitive demands.

### 2.4 Statistical analyses

Data were analyzed using SPSS (Version 29). Scale reliabilities were assessed using Cronbach's alpha. Factor structures were examined using exploratory factor analyses (principal axis factoring with promax rotation), reflecting the adapted nature of the items and their use in a novel task context combining affective arousal and cognitive load indicators. For combined analyses of affective arousal and cognitive load items, a four-factor solution (intrinsic, extraneous, germane load, and affective arousal) was specified *a priori* based on theoretical considerations, to examine whether affective arousal and cognitive load indicators can be empirically differentiated within a shared measurement context. Pre–post differences in affective arousal were analyzed using paired-samples *t*-tests, associations between affective arousal, cognitive load dimensions, and task performance using Pearson correlations, and predictive relations using linear regression analyses. All tests were two-tailed with α = 0.05. Given that the analyses were theoretically guided and focused on a limited set of predefined associations, no formal correction for multiple comparisons was applied. Instead, results were interpreted with appropriate caution, with emphasis placed on the overall pattern and consistency of associations rather than on isolated significance tests (see also Perneger, 1998).

## 3 Results

### 3.1 Affective arousal scale (POET-A)

The pictorial affective arousal scale (POET-A; three items: Balloon, Face, Curve) demonstrated good psychometric properties as a brief online measure of participants' affective arousal. Items were coded on a 0–2 scale, representing under- or over-arousal (0), somewhat low or high arousal (1), and optimal arousal (2), creating a linear index of learning-supportive activation. Pre-task scale means ranged from 1.49 to 1.58 (SDs = 0.61–0.66) and post-task means from 1.59 to 1.63 (SDs = 0.56–0.57), indicating adequate variability. Internal consistency was acceptable to good (Cronbach's α pre = 0.79; post = 0.74), and item-level analyses supported a coherent scale (see Table 2). A paired-samples t-test indicated a small but significant increase in affective arousal from pre- to post-task POET-A (*t*(119) = −2.70, *p* = 0.008, Cohen's *d* = 0.36), suggesting that task engagement slightly elevated affective arousal.

**Table 2:** Item-level descriptives and reliability of the POET-A affective arousal scale.

POET-A item/scale (0-2)	*M*	SD	Corrected r_it	Cronbach's α (if item deleted)
**POET-A pre** (scale mean)	1.53	0.52	—	0.79
Balloon	1.49	0.61	0.69	0.66
Face	1.51	0.66	0.72	0.72
Curve	1.58	0.59	0.77	0.77
**POET-A post** (scale mean)	1.61	0.48	—	0.74
Balloon	1.63	0.56	0.63	0.59
Face	1.59	0.64	0.52	0.67
Curve	1.62	0.57	0.71	0.71

Corrected r_it = corrected item–total correlation; α = Cronbach's alpha (if item deleted).

Convergent and external validity were supported by moderate to strong, highly significant positive correlations between POET-A, the PANAVA arousal subscale, and the post-task verbal arousal rating (Table 3), indicating convergent validity with established self-report measures of subjective activation. Overall, the POET-A scale demonstrated good reliability and validity, providing a solid foundation for subsequent analyses together with cognitive load and task performance.

**Table 3:** Correlations between POET-A, verbal arousal, and PANAVA arousal.

Variable	1	2	3	4
1. POET-A pre	—			
2. POET-A post	0.74^***^	—		
3. Verbal arousal post	0.63^***^	0.61^***^	—	
4. PANAVA arousal	0.62^***^	0.48^***^	0.57^***^	—

POET-A = pictorial affective arousal scale; PANAVA arousal = PANAVA arousal subscale. All correlations are Pearson's r. ^***^p < 0.001.

### 3.2 Cognitive load subscales: intrinsic, extraneous, and germane load

The cognitive load scale showed acceptable to good internal consistency across the three theoretically proposed dimensions. Cronbach's alpha was 0.72 for intrinsic cognitive load, 0.74 for extraneous cognitive load, and 0.78 for germane cognitive load. Corrected item–total correlations ranged from 0.43 to 0.76, across subscales, indicating that all items contributed meaningfully to their respective construct (see Table 4).

**Table 4:** Item statistics and reliability for the cognitive load scale (1–9 coding).

Subscale/Item	*M*	SD	CITC	Cronbach's α (if item deleted)
**Intrinsic load (α = 0.72)**	**3.38**	**1.43**		
1. These tasks were very complex	3.06	1.71	0.49	0.68
2. I invested a lot of mental effort in the complexity of these tasks.	3.68	1.88	0.63	0.51
3. Tasks requiring me to hold two things in mind simultaneously were difficult.	3.39	1.79	0.50	0.68
**Extraneous load (α** **=** **0.74)**	**2.04**	**1.24**		
4. The explanations for these tasks were very unclear.	1.65	1.29	0.63	0.59
5. Unclear explanations required a lot of mental effort.	1.83	1.37	0.61	0.60
6. The task was clearly presented. (rev)	2.66	1.87	0.50	0.79
**Germane load (α** **=** **0.78)**	**6.71**	**1.51**		
7. I was able to maintain focus on the task well.	7.10	1.67	0.72	0.60
8. I was good at concentrating on the task.	7.11	1.71	0.76	0.55
9. I have good strategies to work through such tasks	5.92	2.05	0.43	0.93

Higher scores indicate higher intrinsic or extraneous load; higher germane load indicates more effective cognitive processing. CITC, corrected item–total correlation. Bold values indicate scale means aggregated across the respective items for each subscale.

### 3.3 Exploratory factor analysis: cognitive load and affective arousal

A theory-guided exploratory factor analysis with a four-factor solution specified *a priori* was conducted on all 12 items [three affective arousal (POET-A post) items and nine cognitive load items] to examine structural distinctness. This analysis was designed to assess whether affective arousal and cognitive load indicators can be empirically differentiated within a shared measurement context. At the same time, it provided a secondary validation of the differentiation between cognitive load components, which is noteworthy given that prior research has not always consistently recovered this three-factor structure in subjective load measures.

A four-factor solution corresponding to intrinsic, extraneous, and germane load, as well as affective arousal, was examined using principal axis factoring with Promax rotation. The solution accounted for 56.7% of the variance. All items loaded primarily on their intended factor (pattern loadings ≥0.50), except for the germane load item assessing strategy use (item 9), which showed a weaker yet conceptually consistent loading on the germane factor (0.29) and was retained for conceptual reasons (see Table 5).

**Table 5:** Exploratory factor analysis of cognitive load and affective arousal items (POET-A Post; Four-Factor Solution).

Item	Germane load	Extraneous load	Intrinsic load	Affective arousal
CL_07: Maintained focus	0.93	–	–	–
CL_08: Concentrated well	0.92	–	–	–
CL_09: Used effective strategies^*^	0.29	–	–	–
CL_04: Explanations unclear	–	0.88	–	–
CL_05: Unclear explanations required effort	–	0.76	–	–
CL_06: Task presentation clear (rev.)	–	0.51	–	–
CL_01: Tasks were complex	–	–	0.52	–
CL_02: Mental effort due to task complexity	–	–	0.80	–
CL_03: Hard to keep two things in mind	–	–	0.67	–
Balloon	–	–	–	0.80
Face	–	–	–	0.68
Curve	–	–	–	0.66

Pattern loadings from a principal axis factor analysis with Promax rotation. Only primary loadings ≥ 0.30 are shown. ^*^Item 9 showed a weaker but conceptually consistent loading on the germane load factor.

Inter-factor correlations were moderate (*r* = −0.38 to 0.40), indicating related but distinct constructs. Affective arousal showed a positive association with germane cognitive load (*r* = 0.38) and negative associations with intrinsic (*r* = −0.26) and extraneous cognitive load (*r* = −0.31). These correlations are consistent with the interpretation that affective arousal represents a distinct dimension that complements but is not redundant with cognitive load dimensions.

### 3.4 Relations between cognitive load, affective arousal, and task performance

Associations among cognitive load dimensions, affective arousal, and task performance were examined using Pearson correlations (see Table 6). Post-task affective arousal (POET-A post) was positively associated with germane load and negatively associated with intrinsic and extraneous load.

**Table 6:** Correlations among Stroop-like task performance, cognitive load, and affective arousal.

Variable	1	2	3	4	5	6	7
1. Stroop mean RT	—						
2. Stroop efficiency	−0.61^***^	—					
3. Intrinsic load	0.12	−0.36^***^	—				
4. Extraneous load	0.09	−0.28^**^	0.33^***^	—			
5. Germane load	−0.26^**^	0.26^**^	−0.44^***^	−0.32^***^	—		
6. Affective arousal (pre)	0.07	−0.01	−0.15	−0.11	0.18^*^	—	
7. Affective arousal (post)	−0.16	0.15	−0.23^*^	−0.24^**^	0.32^***^	0.74^***^	—

N = 120. RT = reaction time. Efficiency = number of correct responses divided by RT.

Affective arousal = POET-A (pictorial affective arousal scale). ^*^p < 0.05; ^**^p < 0.01, ^***^p < 0.001.

All cognitive load dimensions were significantly associated with Stroop efficiency, whereas only germane load was additionally linked to faster reaction times, indicating a specific association with attentional control during task execution. Affective arousal scores were not significantly correlated with either reaction time or performance efficiency, indicating that while functional affective arousal aligns with strategic engagement (germane load), it does not show a direct association with immediate task performance.

### 3.5 Pre-task affective arousal and subsequent cognitive load

To examine whether affective arousal prior to task engagement is differentially related to subsequent cognitive load components, linear regression analyses were conducted. Pre-task affective arousal significantly predicted germane cognitive load, *R*^2^ = 0.034, *F*(1,118) = 4.13, *p* = 0.044 (*B* = 0.53, *SE* = 0.26, β = 0.18), whereas no significant associations were observed for intrinsic (*B* = −0.42, *p* = 0.093) or extraneous load (*B* = −0.27, *p* = 0.214).

Together, these findings demonstrate that affective arousal is not uniformly associated with cognitive load components but shows a selective link to germane processing. In contrast to intrinsic and extraneous load, pre-task affective arousal appears to be tied to the quality of strategic, goal-directed engagement rather than to perceived task difficulty or externally imposed strain.

## 4 Discussion

The present study examined affective arousal as a distinct and functionally relevant factor in relation to cognitive load and task performance. Across multiple analyses, affective arousal was consistently and selectively associated with germane cognitive load, while showing weak or negative relations to intrinsic and extraneous load. Notably, pre-task affective arousal predicted subsequent germane cognitive load, but not perceived task difficulty or externally imposed strain.

Although affective arousal was not directly related to immediate task performance, its systematic and selective association with germane processing, as indexed by germane cognitive load items, is consistent with the assumption that affective arousal is linked to how cognitive resources are allocated during task processing.

### 4.1 Affective arousal and cognitive load theory

Within CLT, cognitive demands are traditionally conceptualized in terms of intrinsic load, extraneous load, and germane load, with a primary focus on task structure and instructional design (Sweller et al., 2011). While germane load has been described as cognitive resources devoted to schema construction and strategic processing (Leppink et al., 2013; Sweller, 2010), affective states such as emotion and arousal have generally been treated as peripheral or indirect influences rather than as core components of cognitive processing in CLT (Plass and Kalyuga, 2019).

The present findings align with contemporary interpretations of germane load as a process-oriented construct (Sweller, 2010), showing that affective arousal is selectively associated with germane processing but not or only weak with intrinsic or extraneous load. This selective pattern suggests that arousal does not primarily reflect perceived task difficulty or instructional demands but is more closely linked to learners' strategic and goal-directed engagement during task processing. By empirically differentiating affective arousal from established cognitive load components, the present findings indicate a systematic and selective association with germane processing, highlighting its potential relevance for process-oriented accounts of cognitive resource allocation within CLT.

### 4.2 Germane processing and cognitive resource allocation

Conceptually, the present findings speak to ongoing debates regarding the nature of germane load within CLT. While germane load has often been operationalized via retrospective judgments of perceived learning outcomes (e.g., Leppink et al., 2013), more recent accounts emphasize germane processing as the active investment of cognitive resources driven by learners' decisions rather than externally imposed task demands (Sweller, 2010; see also Klepsch and Seufert, 2021). From this perspective, germane processing is not defined by what is learned, but by how learners allocate available cognitive resources.

The present operationalization of germane cognitive load captures process-related aspects of cognitive engagement, such as sustained attention, concentration, and strategy use, consistent with a process-oriented interpretation of germane processing. While conceptually related to engagement, these aspects are interpreted more specifically as indicators of cognitive resource allocation during task performance, reflecting how germane processing manifests at the level of subjective experience.

The present study adopts this process-oriented view by focusing on attentional engagement, concentration, and strategic regulation during task execution. This distinction is particularly relevant for interference-rich tasks such as Stroop paradigms, where learning outcomes are limited but sustained attentional control and strategic engagement are central to efficient task performance. Accordingly, in the present study, germane processing in such tasks is understood in terms of learner-driven resource allocation rather than outcome-based learning gains.

### 4.3 Selective association of arousal with germane processing

A central contribution of the present study lies in the selective nature of the observed associations between affective arousal and cognitive load components. Across analyses, affective arousal was consistently linked to germane cognitive load, while showing negative or near-zero relations with intrinsic and extraneous load. This dissociation is theoretically meaningful, as intrinsic and extraneous load primarily reflect task-inherent complexity and instructional design rather than learner-driven processing. In contrast, germane load captures the extent to which available cognitive resources are actively invested in strategic, goal-directed processing.

The present findings therefore suggest that affective arousal is not a general marker of cognitive burden but is specifically associated with how learners allocate resources toward productive processing. Notably, the predictive role of pre-task arousal for subsequent germane load further supports this interpretation, indicating that affective activation may orient learners toward deeper engagement before task demands are fully encountered. Importantly, the interpretation is not based on the magnitude of individual correlations but on the systematic pattern of associations across load components, which consistently differentiates germane processing from intrinsic and extraneous load. Although the observed associations are modest in magnitude, their consistent direction across analyses supports the interpretation of a selective relation between affective arousal and germane processing.

### 4.4 Toward an affective resource allocation perspective

Taken together, the present findings are most coherently interpreted within an affective resource allocation perspective on cognitive processing in learning tasks. From this viewpoint, affective arousal does not constitute an additional source of cognitive load but rather can be understood as a contextual factor that shapes the allocation of available cognitive resources. Moderate, learning-relevant arousal appears to orient learners toward strategic, goal-directed engagement, thereby facilitating germane processing (as reflected in germane load ratings) while remaining largely independent of task-inherent complexity or externally imposed demands. This perspective is compatible with classic assumptions about arousal and performance (Yerkes and Dodson, 1908) yet extends them by embedding affective activation within the framework of CLT. Rather than exerting a direct influence on performance outcomes, affective arousal may influence the quality of cognitive processing by shaping attentional focus and strategic investment. In this sense, affective arousal can be understood as a boundary condition under which germane processing is more or less likely to occur.

### 4.5 Limitations and future directions

The present findings should be interpreted in light of several methodological and conceptual considerations. First, although the germane cognitive load scale showed good internal consistency, one item capturing strategy-related engagement (CL09) exhibited a weaker factor loading. This may reflect the conceptual heterogeneity of germane processing, which has been described as encompassing effortful schema construction, strategic regulation, and sustained task engagement (Sweller, 2010; see also Klepsch et al., 2017). In the present study, strategy endorsement may therefore reflect a complementary but partly distinct facet of cognitive engagement compared to sustained attentional focus and concentration. Future research may benefit from further differentiating facets of germane processing, for example by employing larger item sets or combining self-report measures with process-based indicators.

Second, the germane load items were adapted rather than adopted verbatim from established instruments (Leppink et al., 2013). While this limits direct comparability with prior studies, it aligns with contemporary interpretations of germane processing.

Third, affective arousal was operationalized using a three-level coding scheme distinguishing functionally unfavorable extreme under- or over-arousal, moderate deviations from optimal arousal, and an optimal, learning-conducive arousal state. While this theoretically motivated coding captures functional differences in arousal, it may limit sensitivity to more fine-grained variations in arousal intensity. Future research may therefore examine whether broader or more continuous arousal measures provide additional resolution in capturing engagement-related processes.

Finally, the use of a brief Stroop-like task limits conclusions regarding longer-term learning processes. Extending the present approach to more complex learning environments but also to longitudinal and experimental designs will be important to further examine how affective arousal shapes cognitive resource allocation in different complex learning tasks and over time. Future research may further clarify the causal role of affective arousal in germane processing by examining systematic variations in learners' activation states and their relation to patterns of cognitive engagement. By relating such variations to systematic differences in task characteristics this will furthermore enable closer connections to established CLT paradigms.

## 5 Conclusion

The present study shows that affective arousal is a distinct and functionally relevant factor in cognitive processing, selectively supporting germane processing while remaining largely independent of task-inherent difficulty and extraneous demands. Rather than reflecting cognitive burden, affective arousal appears to be associated with how available cognitive resources are allocated during task engagement. Taken together, these findings support an affective resource allocation perspective, according to which momentary affective arousal functions as a regulatory condition shaping the quality of cognitive engagement. From this perspective, germane processing reflects how learners actively mobilize cognitive resources as a function of their momentary activation state.

## Data Availability

The raw data supporting the conclusions of this article will be made available by the authors, upon reasonable request.
